# Effects of Acupuncture-Combined Tuina on Patients With Knee Osteoarthritis: Protocol for a Randomized Controlled Trial

**DOI:** 10.2196/84082

**Published:** 2026-02-05

**Authors:** Chendong Gu, Dong Wei, Kaiyue Zhang, Jinxi Ren, Yihui Qiao, Fengyu Zhang, Yiting Zhu, Zhixian Xu, Yinlong Cao, Shuyi Yao, Hanrui Guo, Yihang Wang, Huan Wang, Lin Wang, Quanliang Wang, Guangxin Guo

**Affiliations:** 1 Department of Acupuncture and Moxibustion Massage, Qingpu Traditional Chinese Medicine Hospital, Shanghai, China; 2 Department of Tuina, Shanghai Municipal Hospital of Traditional Chinese Medicine, Shanghai University of Traditional Chinese Medicine, 274 Zhijiang Middle Road, Jing'an District, Shanghai, Shanghai, 200071, China, 86 18621831323; 3 School of Acupuncture-Moxibustion and Tuina, Shanghai University of Traditional Chinese Medicine, Shanghai, China; 4 School of Integrative Medicine, Shanghai University of Traditional Chinese Medicine, Shanghai, China; 5 Yueyang Clinical Medical College, Shanghai University of Traditional Chinese Medicine, Shangai, China; 6 School of Traditional Chinese Medicine, Shanghai University of Traditional Chinese Medicine, Shanghai, China; 7 The Third Affiliated Hospital of Henan University of Traditional Chinese Medicine, Henan, China; 8 Shuguang Hospital, Shanghai University of Traditional Chinese Medicine, Shanghai, China; 9 Department of Traditional Chinese Medicine, Clinical Medicine, Chongqing University School of Medicine, Chongqing, China; 10 Shanghai Pudong New Area People's Hospital, Shanghai, China; 11 Department of Acupuncture-Moxibustion and Tuina, Henan Integrative Medicine Hospital, Zhengzhou, China

**Keywords:** knee osteoarthritis, Tuina, acupuncture, pain, stiffness, multimodal magnetic resonance imaging

## Abstract

**Background:**

Knee osteoarthritis (KOA) is a prevalent degenerative joint disease that is characterized by joint pain, stiffness, and dysfunctional impairment, imposing a substantial medical burden annually. Tuina, a widely used noninvasive traditional Chinese medicine therapy commonly used for KOA, has been used for the management of this condition; however, its additive benefit to acupuncture remains unclear.

**Objective:**

The objective of the study is to assess whether the effectiveness of acupuncture-combined Tuina is superior to that of acupuncture in the management of KOA.

**Methods:**

This single-center, parallel-group, randomized controlled trial aims to enroll 60 patients with KOA, who will be randomly assigned to either a Tuina group (acupuncture-combined Tuina) or an acupuncture group (30 patients per group). Baseline assessments will include demographic and clinical evaluations: the visual analog scale from the short-form McGill Pain Questionnaire, pain threshold, muscle tension, 10-meter walking test, Western Ontario and McMaster Universities Osteoarthritis Index, and multimodal magnetic resonance imaging. Over a 6-week intervention, the Tuina group will receive Tuina on the basis of acupuncture, that is, acupuncture-combined Tuina, while the acupuncture group will undergo just acupuncture, with both groups continuing standard care as prescribed. Posttreatment, clinical outcomes and safety will be reassessed using baseline indicators. A 12-week follow-up will include all clinical evaluations. Assessments will be conducted by blinded assessors, and statistical analyses will be conducted by independent, blinded analysts. Outcomes will evaluate clinical pain and functional differences between groups and will elucidate the underlying cerebral mechanisms.

**Results:**

This study was funded in August 2024. The experimental plan will begin on December 31, 2025, and end on May 4, 2027.

**Conclusions:**

This trial aims to verify whether acupuncture-combined Tuina receives better efficacy than single acupuncture, as well as to explore the neuroimaging mechanisms that are clinically affected, thus providing scientific evidence for the treatment of clinical patients with KOA.

## Introduction

Knee osteoarthritis (KOA) is a prevalent and debilitating condition characterized by joint pain, stiffness, and restricted mobility, significantly impairing daily functioning and work participation [1-3]. In the United Kingdom, employees with arthritis have a 1.35% higher rate of sickness absence than their counterparts without arthritis [4]. The prevalence of KOA is 8.1% in China and 12.1% in the United States and Europe, with rates expected to rise as global populations age [5,6]. Currently, there is no treatment that can completely stop or reverse the progression of osteoarthritis [7,8]. Due to painfulness from the knee joint, patients usually use painkillers, but there is a risk of overuse and side effects of analgesic drugs [7-9]. The effectiveness of acupuncture in treating KOA in improving pain has been preliminarily verified [10,11]. Tuina, a traditional Chinese medicine manipulation therapy, is applied in the clinical treatment of KOA. However, whether the clinical efficacy of acupuncture-combined Tuina is more effective than single acupuncture remains to be proven.

Recent advances in neuroimaging, particularly the use of multimodal magnetic resonance imaging (multi-MRI), have provided powerful tools for investigating changes in brain activity involved in the pathogenesis of KOA, complementing research on biomechanics, aging, and genetic factors [12-14]. Pain, the hallmark symptom of KOA, results from complex interactions between brain regions responsible for pain transmission and modulation [15-17]. Emerging evidence suggests that KOA-associated pain is linked to functional activation and structural alterations in the cerebral cortex and subcortical regions [18,19]. Studies have identified increased resting-state functional connectivity between the ventrolateral periaqueductal gray and the bilateral thalamus in patients with KOA with knee pain compared to healthy controls [20]. Additionally, gray matter (GM) volume reductions in the bilateral insula and hippocampus have been observed [21]. Both static and dynamic functional network connectivity have been shown to correlate with clinical symptoms in KOA [22], highlighting the complex neural mechanisms underlying the condition.

Acupuncture, a core component of traditional Chinese medicine, is recommended in the Chinese Guidelines for Diagnosis and Treatment of Osteoarthritis (2024 Edition) as an effective treatment for alleviating pain and improving joint function in patients with KOA [23]. Studies have demonstrated that acupuncture effectively reduces joint pain and enhances functionality [24,25], with its therapeutic benefits lasting up to 4.5 months compared to sham acupuncture [26]. Evidence suggests [27-30] that acupuncture alleviates KOA by modulating central neural pathways, with its mechanisms varying based on the treatment modality and patient pathology, as reflected in specific cerebral alterations and activation patterns.

Tuina, a noninvasive Chinese therapeutic massage, is widely used for managing KOA due to its direct effects on the musculoskeletal system [31]. Through techniques such as pressing and kneading, Tuina alleviates pain and stiffness, while also improving knee stability, muscle strength, and joint mechanics, thus supporting long-term recovery [23]. Unlike acupuncture, which focuses on neurophysiological pathways, Tuina emphasizes physical manipulation of the musculoskeletal system to address knee joint dysfunction and muscle tension, enhancing joint stability and optimizing movement patterns [32,33]. The process of Tuina tends to be more acceptable to patients, as it is tender, comfortable, and associated with no injury, which is able to effectively relieve the pain, negative emotion, and dysfunction in patients with KOA.

As a result, to compare whether acupuncture-combined Tuina is better than single acupuncture, as well as to reveal the mechanism of analgesic effect that acupuncture-combined Tuina imposes to the brain center, an optimal, efficient, randomized comparative trial will be carried out, aiming to provide more scientific evidence for treating patients with KOA clinically.

## Methods

### Design

This is a single-center, parallel, randomized controlled trial. On the basis of the criteria established by the American College of Rheumatology [34], a total of 60 participants diagnosed with KOA will be recruited as eligible participants. These participants will be randomly assigned to 2 groups, each consisting of 30 individuals: the Tuina group (acupuncture-combined Tuina) and the acupuncture group. The intervention duration for both groups will be 30 minutes, administered 2 times weekly. The treatment period will last for 6 weeks, followed by a 12-week follow-up period.

**Figure 1.**
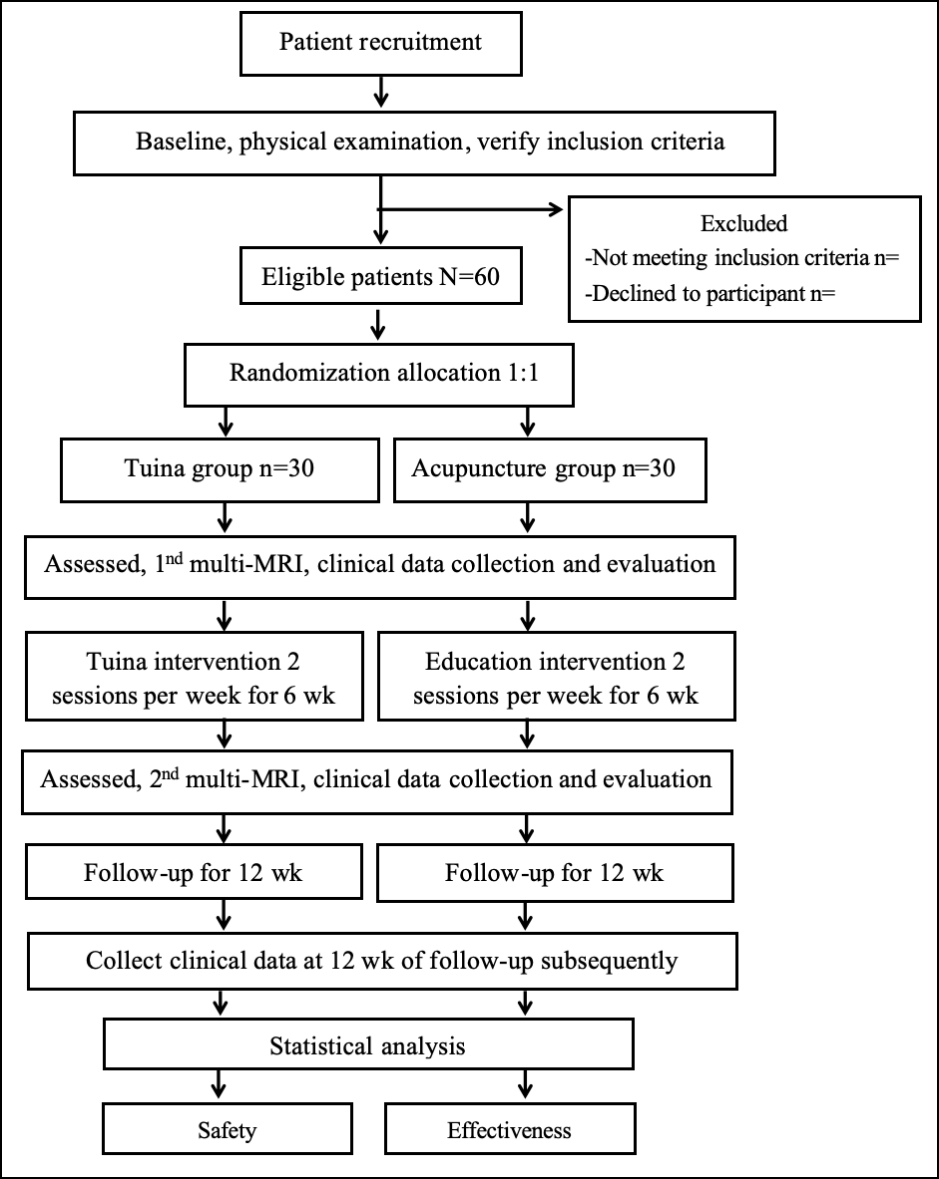
Flowchart of the trial. This study is a randomized controlled trial using multimodal magnetic resonance imaging (multi-MRI) and plans to enroll 60 patients with knee osteoarthritis. These patients will be equally allocated to 2 groups: the Tuina group and the acupuncture group, with 30 patients in each group. The trial will include a 6-week treatment period. During the treatment, patients in the Tuina group will receive 12 Tuina sessions based on acupuncture, whereas those in the acupuncture group will only receive acupuncture. Outcome assessments and multi-MRI scans will be conducted at 2 time points: baseline and the end of the treatment (6 weeks later). Subsequently, only clinical outcomes will be evaluated during the 12-week follow-up period.

Outcome measurements and magnetic resonance imaging scans will be assessed at baseline and at 6 weeks posttreatment, followed by a 12-week clinical follow-up period. Once data collection is completed, analyses will be conducted to evaluate changes in clinical indices and cerebral multi-MRI parameters across groups. The trial flowchart and study schedule for data collection are illustrated in Figure 1 and Table 1, respectively.

**Table 1.** Study schedule for data collection^a^.

Period	−2 weeks	0 weeks	0‐6 weeks	6 weeks	6‐18 weeks	18 weeks
	Screening	Baseline	Intervention	Assessing	Follow-up	Assessing
Enrollment						
Eligibility	✓^b^					
Demography	✓					
Informed consent	✓					
Sign the informed consent		✓				
Medical history	✓		✓	✓	✓	✓
Physical examination	✓		✓	✓	✓	✓
Randomization		✓				
Interventions						
Tuina group (n=30)			✓			
Acupuncture group (n=30)			✓			
Assessments						
VAS^c^	✓	✓		✓		✓
SF-MPQ^d^	✓	✓		✓		✓
Pain threshold		✓		✓		✓
Muscle tension		✓		✓		✓
10MWT^e^		✓		✓		✓
WOMAC^f^		✓		✓		✓
Clinical efficacy				✓		✓
Multi-MRI^g^		✓		✓		
Trial evaluation						
Patients’ compliance				✓		✓
Safety evaluation				✓		✓
Credibility test				✓		✓
Adverse events				✓		✓
Analysis		✓	✓	✓	✓	✓

a The informed consent and examination will be conducted after recruitment. Then, matched patients with knee osteoarthritis will be randomized into 2 groups and receive treatment. Both clinical outcomes and multimodal magnetic resonance imaging scans will be performed at 2 time points. Only clinical data will be collected at the 12-week follow-up subsequently. Adverse events will be recorded in the case report form at any time during the study.

b ✓: used to indicate that the inspection item needs to be executed at the corresponding time point.

c VAS: visual analog scale.

d SF-MPQ: short-form McGill Pain Questionnaire.

e 10MWT: 10-meter walking test.

f WOMAC: the Western Ontario and McMaster Universities Osteoarthritis Index.

g Multi-MRI: multimodal magnetic resonance imaging.

### Participants

All patients will be recruited from the Qingpu Traditional Chinese Medicine Hospital, Shanghai, through a combination of posters, online announcements, and promotional pamphlets. The inclusion and exclusion criteria for participants are presented in Textbox 1.

Textbox 1. **The inclusion and exclusion criteria for participants.**

Inclusion criteria

- Meet the diagnostic criteria for knee osteoarthritis set by the American College of Rheumatology [34].
- Be aged between 40 and 70 years, with left knee pain mainly or left knee pain only, as well as right-hand dominance.
- Have a Kellgren-Lawrence radiological [35] grade of I-II.
- Present with a visual analog scale score >3 from the short-form McGill Pain Questionnaire and a disease duration of at least 3 mo.
- Voluntarily agree to participate in this study and sign the informed consent form.

Exclusion criteria

- Have a history of trauma or knee surgery.
- Have tumors, tuberculosis, osteomyelitis, or other diseases affecting the knee.
- Exhibit severe hepatic or renal dysfunction, severe cardiovascular diseases, diabetes mellitus, or mental illnesses that may interfere with the treatment of Tuina.
- Experience pain in the knee area or throughout the body due to other diseases.
- Have magnetic resonance imaging (MRI) contraindications, such as claustrophobia, pacemakers, defibrillators, heart stents, or intrauterine devices or any other conditions that prevent safe MRI scanning.
- Have skin lesions in the periarticular area of the knee joint.

### Sample Size Calculation

This study is designed as a superiority trial, with the sample size calculated using PASS 15 (NCSS, LLC). On the basis of relevant literature, a 2-point difference on the visual analog scale (VAS) score was defined as the superiority margin [36]. According to our preliminary experiments, the mean difference between the acupuncture group and the Tuina group (acupuncture-combined Tuina) is 3.25, with an SD of 1.5 in both groups. Using a superiority test with an *α* of .025 and a power of 0.8, and accounting for a 20% dropout rate, the calculated sample size is 30 cases per group, resulting in a total of 60 cases required.

### Randomization and Allocation Concealment

This study uses blocked randomization to ensure unbiased group allocation. The statistical software SPSS 21.0 will be used to randomly assign participants to either the Tuina group or the acupuncture group. The randomization process will be performed by an independent statistician who will generate 60 random numbers of varying sizes using a computer. This statistician will have no involvement in participant recruitment, assessment, treatment administration, or data analysis, ensuring the independence and impartiality of the process.

Participants will be sequentially numbered from 1 to 60, and blocked randomization will be applied with a block size of 4. Within each block, treatment allocation (Tuina or acupuncture) will be determined by computer-generated random numbers, with smaller numbers assigned to Tuina and larger numbers assigned to acupuncture. The allocation process within each block will be dynamic and unpredictable, ensuring an equal distribution of participants between the 2 groups in a 1:1 ratio.

To ensure allocation concealment, the randomization scheme will be enclosed in opaque, sealed envelopes, numbered sequentially according to participant enrollment. Each envelope will contain the allocation result for the corresponding participant, whereas the outside will remain blank to prevent unintentional disclosure. These envelopes will remain sealed until the treatment allocation is determined, and they will be stored securely to prevent premature access to the allocation information. The assigned treatment (acupuncture-combined Tuina group or the acupuncture group) will be revealed only by opening the envelope corresponding to the participant’s number, ensuring the integrity of allocation concealment and enhancing the scientific rigor of the study.

### Blinding

This clinical trial will implement blinding for assessment to minimize potential biases. Statistical analysts will be blinded to participant recruitment, allocation, and treatment details to ensure the integrity and reliability of the study results. They will not have access to raw data until after the data collection process is completed and quality control measures are in place.

Due to the nature of Tuina and acupuncture interventions, achieving full blinding of patients and therapists is not feasible [37]. To minimize potential bias, therapists will not be involved in participant information collection, assessment, or subsequent data analysis. They will also refrain from disclosing information regarding the specific benefits or risks of the treatments to patients [38]. Additionally, to mitigate bias, outcome assessors and statisticians will remain blinded to group allocation throughout the study.

### Interventions

#### Overview

Participants in this study will receive a total of 12 treatment sessions over a 6-week period. The interventions will be administered by therapists with at least 6 years of professional experience in Tuina and acupuncture. All therapists will undergo standardized training to ensure consistency in treatment delivery, focusing on session duration, target treatment areas, and the specific techniques used.

To maintain the study’s integrity, patients will be prohibited from using concomitant medications or receiving alternative interventions during the treatment period. Celecoxib (Pfizer) will be allowed for pain management, but only if necessary and not within 48 hours before outcome measurements. During the follow-up phase, any medication use or additional interventions will be documented thoroughly to ensure an accurate assessment of the effects of Tuina and acupuncture, thereby isolating their impact on patient outcomes. This approach will guarantee the validity and reliability of the study findings.

#### Acupuncture Group

In this study, acupuncture treatment will follow a standardized protocol based on traditional Chinese medicine principles, expert consensus, and clinical experience [39]. Participants will receive acupuncture in a private room, with the affected knee being the sole treatment focus for those with osteoarthritis symptoms. Sterile, single-use needles (0.25 mm gauge, 25‐40 mm in length, Hwato) will be used.

**Figure 2.**
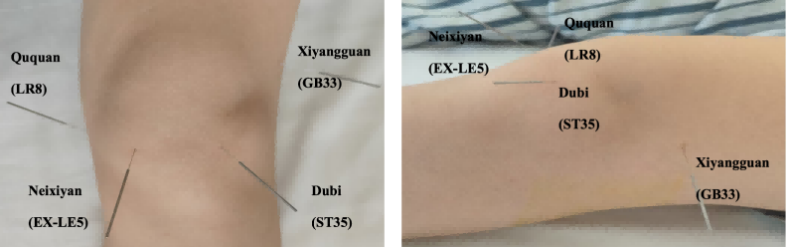
Location of acupoints for the acupuncture group. Core acupoints: Dubi (ST35), Neixiyan (EX-LE5), Ququan (LR8), and Xiyangguan (GB33).

Before the study, all acupuncturists will undergo comprehensive training on standardized procedures, including precise acupoint location and needle manipulation techniques. The acupuncture protocol will involve five core acupoints: Dubi (ST35), Neixiyan (EX-LE5), Ququan (LR8), Xiyangguan (GB33), and an Ashi point, with up to 3 supplementary points chosen based on the affected meridian (Figure 2). Ashi points are defined as tender points within the knee joint region, marked by sensations of soreness, distension, or pain.

Achieving the De qi sensation, a combination of soreness, numbness, distension, and heaviness, will be required for every session. Participants will receive two 30-minute acupuncture sessions per week for 6 weeks, totaling 12 sessions. Sessions will be spaced at least 48 hours apart.

#### Tuina Group

##### Overview

Participants randomized to the Tuina intervention arm will undergo integrated acupuncture and Tuina in private treatment settings, following the same standardized acupuncture protocol as that administered in the acupuncture group. The Tuina protocol will include manual therapy, covering Tuina techniques applied to both sides of the affected limb, as well as passive range-of-motion exercises for the knee joint [40,41].

To ensure consistency and reproducibility across all sessions and practitioners, the core Tuina manipulations (rolling, kneading, and pressing) are formulated primarily according to the *Tuina Therapy–14th Five-Year Plan Textbook* and previous research and will be executed with a fixed order listed below.

##### Step 1: Rolling in Supine Position

The practitioner’s right hand should exert force on the dorsal ulnar side, stick it to the anterior thigh, and roll back and forth through continuous movement of wrist flexion and extension and forearm pronation and supination. The frequency will be approximately 120 times per minute. The manipulation will be repeated from top to bottom 3 to 5 times [42].

##### Step 2: Kneading and Pressing Technique

This step focuses on the application of kneading and pressing techniques.

- Kneading: use the left or right thrusting thread or the fingertip of the thumb to perform rhythmic spiral movements.
- Pressing: using their thumb or index finger, the physician should press the corresponding acupoints with the middle finger or index finger for 5 to 10 seconds.

Both kneading and pressing techniques will be applied to Heding (EX-LE2), Neixiyan (EX-LE4), Dubi (ST35), Yanglingquan (GB34), Yinlingquan (SP9), Xuehai (SP10), and Ashi points, taking the patient’s feeling of soreness, distension, and pain as the degree of tolerance without exerting too much force, aiming to elicit the De qi sensation, which is indicative of effective manipulation. Following this, patients will transition to a prone position.

##### Step 3: Rolling in Prone Position

**Figure 3.**
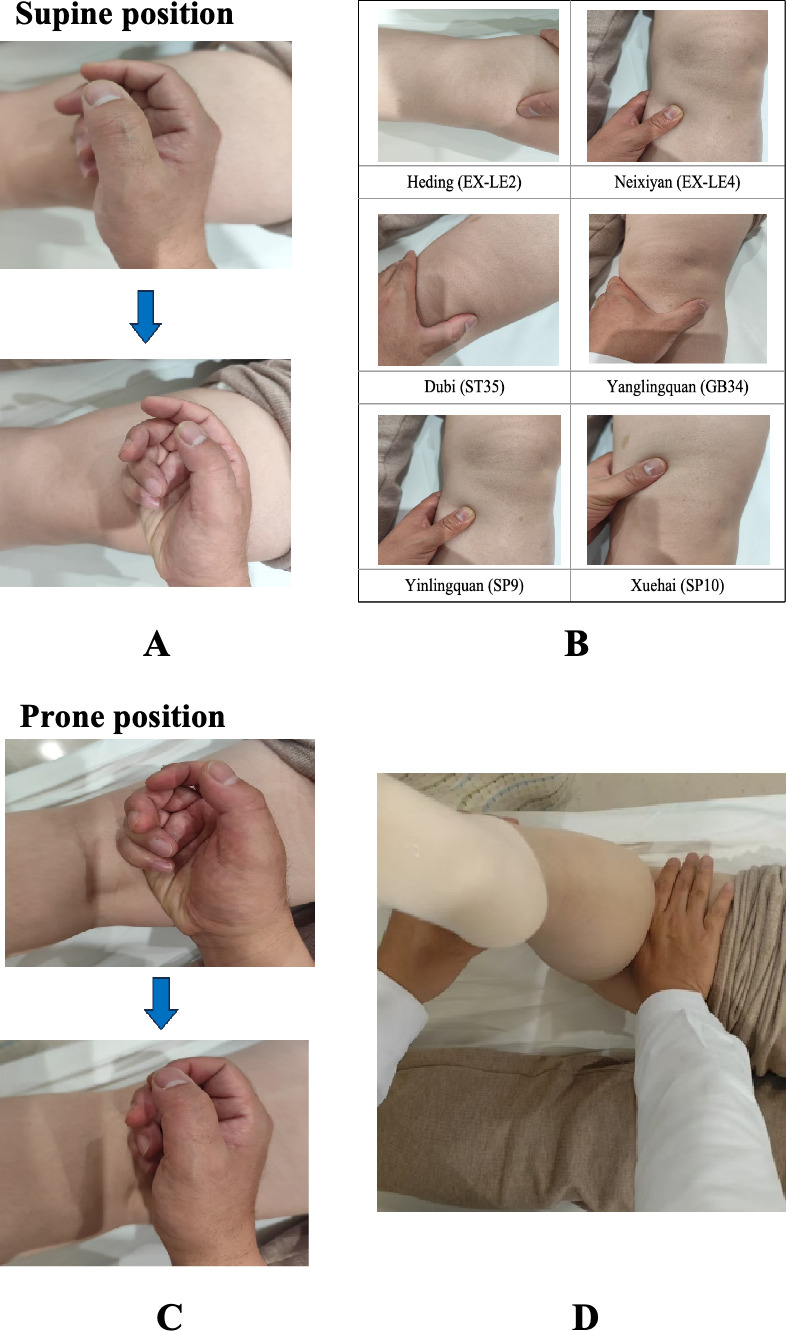
Demonstration of Tuina. (A) Initially, patients will be positioned supine, with the practitioner standing beside them. The practitioner will use the back of the hand to perform rolling motions over the anterior thigh. (B) Subsequently, kneading and pressing techniques will be applied to key acupoints, including Heding (EX-LE2), Neixiyan (EX-LE4), Dubi (ST35), Yanglingquan (GB34), Yinlingquan (SP9), Xuehai (SP10), and Ashi points. (C) The practitioner will then use the back of the hand to perform rolling motions over the posterior thigh, popliteal fossa, and posterior leg. (D) Finally, one hand will be used to apply pressure to the knee joint, while the other hand holds the ankle joint to facilitate passive flexion and extension exercises for the lower limbs for 1 min.

The practitioner will then use the back of the hand to perform rolling motions over the posterior thigh, popliteal fossa, and posterior leg. The operational standard of rolling is identical to the rolling technique in step 1 (Figure 3). This sequence aims to relax the muscles and stimulate the collaterals.

##### Step 4: Kneading and Passive Joint Manipulating

This step combines kneading with passive joint manipulation.

1. Kneading: The thumb will be used to apply pressure from the posterior thigh to the knee joint, whereas the other 4 fingers and the palm help stabilize the thigh.
2. Passive joint manipulating: When one hand is manipulating the kneading technique, the other hand holds the ankle joint to facilitate passive flexion and extension exercises for the lower limbs for 1 minute.

Kneading and pressing techniques will be applied to Weizhong (BL40) and Chengshan (BL57). In total, participants will receive two 30-minute sessions per week for 6 weeks, totaling 12 sessions, with a minimum interval of 48 hours between treatments.

### Follow-Up

Upon completion of the 6-week intervention period, all patients will immediately transition into a 12-week unsupervised follow-up phase. At the end of the 18-week clinical period, patients with KOA will undergo assessments for all clinical evaluations, with the exception of except for the multi-MRI outcomes.

### Multi-MRI Examination Procedure

Multi-MRI data will be acquired using a 3.0-T magnetic resonance scanner (SIEMENS MAGNETOM Verio syngo MR B17), equipped with a 32-channel phase-array head coil, at the Institute of Science and Technology for Brain-Inspired Intelligence, Fudan University.

Before the magnetic resonance imaging (MRI) scan, participants will undergo a pre-MRI evaluation procedure, which includes instructions to rest for 10 minutes, relax, and maintain calmness throughout the session. During the scan, participants will be requested to close their eyes, wear earplugs, keep their heads stationary, remain relaxed and awake, and avoid thinking of anything specific.

The imaging protocol will include the following steps:

- High-resolution T1-weighted image: A spin-echo sequence will be used to acquire a transverse sagittal scan with a flip angle of 9°, a repetition time (TR) of 1900 ms, an echo time (TE) of 2.93 ms, a field of view of 256×256 mm², 160 slices, and a slice thickness of 1 mm.
- Diffusion tensor imaging (DTI): DTI will be performed using axial DTI mapping sequences with a TR of 10,000 ms, a TE of 89 ms, a matrix size of 240×240, a slice thickness of 2 mm, B-values of 0 and 1000 s/mm², and 30 directions.
- Blood oxygenation level–dependent resting-state functional imaging: An echo-planar imaging sequence will acquire blood oxygenation level dependent–resting-state functional images with a coronal axial scan, 33 slices, a slice thickness of 4 mm, a TE of 30 ms, a TR of 2000 ms, a field of view of 220×220 mm², a voxel size of 3.4×3.4×4.0 mm³, a flip angle of 90°, and a scan duration of 8 minutes and 8 seconds, resulting in 240 volumes.

Following the MRI procedure, participants will be given an additional 5-minute rest period and may leave if they experience no discomfort.

### Outcome Measurements

#### Assessment Tools and Schedule

Clinical outcomes will be evaluated using 2 self-report questionnaires, 4 instrument-based indicators measured at baseline and after 6 weeks of treatment, and multi-MRI data. At the conclusion of the full 18-week clinical period, all clinical assessments, excluding multi-MRI outcomes, will be performed. The responses will provide valuable information on pain levels, sensory experiences, emotional states, and overall quality of life.

#### Primary Outcomes

The VAS score is based on a 10-cm long straight line, with the starting end marked as 0 points, indicating no pain, and the end marked as 10 points, indicating unbearable severe pain. Patients will select a point on the line that represents their current level of pain. A lower total score indicates reduced pain severity, reflecting the effectiveness of the interventions. This VAS is distinct from the VAS component within the short-form McGill Pain Questionnaire (SF-MPQ), which is used solely for screening purposes and for baseline characterization.

#### Secondary Outcomes

##### Short-Form McGill Pain Questionnaire

The SF-MPQ will be used to assess the multidimensional experience of pain. It consists of 2 primary components.

###### Descriptive Pain Rating

This component comprises a list of 15 pain descriptors, categorized into 11 items evaluating sensory qualities of pain (eg, throbbing and shooting) and 4 items evaluating affective qualities (eg, tiring and fearful). Each descriptor is rated on an intensity scale ranging from 0 (none) to 3 (severe). The scores are summed to yield a sensory score (range 0‐33), an affective score (range 0‐12), and a total pain rating index (range 0‐45).

###### Present Pain Intensity

The present pain intensity is a single-item, 6-point verbal rating scale (0=no pain, 5=excruciating) that provides a global assessment of current pain intensity [43].

The pain intensity scores derived from the SF-MPQ will not be used as outcome measures in the longitudinal analysis.

### Pain Threshold

Pain threshold will be assessed at specific knee points—EX-LE02 (Heding), EX-LE04 (Neixiyan), EX-LE05 (Xiyan), and the area beneath the tip of the patella—using a pressure algometer. This measure will evaluate the sensitivity to mechanical pressure pain in patients with KOA [44,45].

### Muscle Tension

Muscle tension will be assessed in the quadriceps and gastrocnemius muscles, providing insights into muscle stiffness in patients with KOA [46].

### 10-Meter Walking Test

The 10-meter walking test (10MWT) will assess walking speed by timing the patients over a 10-m distance, with a focus on the middle 6 m to account for acceleration and deceleration. The test will begin when the toe of the leading foot crosses the 2-m mark and will end when the toe crosses the 8-m mark. Three trials will be conducted, and the average time will be recorded.

### Western Ontario and McMaster Universities Arthritis Index

The Western Ontario and McMaster Universities Arthritis Index (WOMAC) questionnaire will evaluate symptoms of KOA in 3 domains: pain (5 questions), stiffness (2 questions), and functional limitations during daily activities (17 questions) [47].

### Clinical Efficacy

Clinical efficacy will be categorized into 4 levels: cure, markedly effective, effective, and ineffective [48].

- Cure: complete resolution of pain and symptoms, with normal joint activity
- Markedly effective: resolution of pain and symptoms, with no limitation in joint activity
- Effective: significant reduction in pain and symptoms, with slight joint activity limitation
- Ineffective: no significant improvement in pain, symptoms, or joint activity

### Multi-MRI Outcomes

#### Functional Imaging

Functional connectivity analysis will be used to assess brain network activity and interregional communication, highlighting potential neural mechanisms underlying pain perception and motor function in KOA.

#### Diffusion Imaging

Fractional anisotropy and mean diffusivity will be measured to assess white matter (WM) integrity, providing insights into neural pathway alterations in patients with KOA.

#### Structural Imaging

GM density, cortical thickness, and volumes of subcortical nuclei will be evaluated to investigate brain structural changes related to KOA.

### Safety Evaluation and Adverse Events

Participant safety will be actively monitored throughout the trial. At each treatment session and assessment visit, practitioners will systematically inquire about and record any adverse events (AEs). The following treatment-related AEs will be proactively monitored:

- For acupuncture: significant needle pain, bleeding, hematoma or bruising (>2 cm), dizziness or fainting, and local infection [49]
- For Tuina: transient (>24 h) increases in pain, significant bruising, skin irritation, and unexpected joint discomfort [41]
- General: any other unexpected AE reported by the participant [42]

All AEs will be recorded in the case report form (CRF) with details on severity, duration, action taken, and outcome. The causality relationship to the intervention will be assessed by the investigator. The incidence and type of AEs will be summarized and compared between the 2 treatment groups as a key safety outcome. All AEs will be managed, recorded, and reported in accordance with the study protocol and the requirements of the Ethics Committee.

### Data Management

Baseline data will be collected using CRFs, which will include clinical observations, outcome measures, AEs, and safety evaluations. Outcome assessors, who are independent of the data management team, will ensure impartiality in evaluations, completing forms based on anonymous participant identifiers to maintain blinding. Completed CRFs will be entered into a Microsoft Excel database by 2 independent data administrators who are blinded to group allocations and trained in data monitoring. An independent data manager will oversee data entry into the International Traditional Medicine Clinical Trial Registry.

Data will be stored securely in both physical and digital formats, with access limited to the data manager. Upon trial completion, anonymization procedures will be implemented to preserve participant confidentiality, and an independent data analyst will perform statistical analysis on the anonymized data, ensuring objectivity and transparency.

### Quality Control

Rigorous quality control measures will be enforced by the steering committee. All researchers will undergo training in trial methodologies and monitoring techniques before participation. Any changes to the study protocol will be communicated to both the steering and ethics committees to ensure adherence to the original plan. The committees will also oversee recruitment, the intervention protocols (Tuina and acupuncture), and the quality of data collection.

### Statistical Analysis

All statistical analyses will be conducted using IBM SPSS 27.0 and R software (version 4.3.0), with a 2-sided *P* value of <*.05* considered statistically significant. Analyses will commence after the completion of follow-up for the last participant and will adhere to the intention-to-treat principle.

Changes in the primary outcome (VAS) and key secondary outcomes (WOMAC, 10MWT, pain threshold, and muscle tension) over time will be analyzed using linear mixed-effects models. These models will directly test whether the pattern of change from baseline through the 6-week and 18-week assessments differs between the Tuina and acupuncture groups (ie, a group×time interaction). If this interaction is significant, post hoc tests (ie, Bonferroni) will be conducted to identify the time points at which the groups differ.

The mixed-effects models appropriately handle missing data under the assumption that they are missing at random. A sensitivity analysis using multiple imputation will also be conducted to confirm the robustness of the primary results. Baseline characteristics will be summarized using means (SDs) or medians (IQRs). Differences between the 2 groups at baseline will be tested using *t* tests or Mann-Whitney *U* tests for continuous variables and chi-square tests for categorical variables. Correlations between changes in different outcomes will be assessed using Pearson or Spearman coefficients.

### Analysis of Multi-MRI Data

Structural MRI data will be processed using FSL tools (FMRIB Software Library). SIENAX will calculate normalized volumes of neocortical GM, total GM, and WM [50], while subcortical region volumes (eg, hippocampus, thalamus) will be estimated using FMRIB’s integrated registration and segmentation tool [51]. The cortical thickness will be measured at each vertex using FreeSurfer [52].

The DTI pipeline will follow FSL’s diffusion toolbox to generate fractional anisotropy and mean diffusivity images, with voxelwise tract-based spatial statistics applied for group comparisons. Resting-state functional magnetic resonance imaging data preprocessing and functional connectivity analyses will be performed using SPM12 software within MATLAB 2013b (MathWorks). This rigorous, standardized approach ensures reliability and accuracy in statistical and neuroimaging analyses.

### Patient and Public Involvement

The design, conduct, reporting, and dissemination plans of this study will be carried out without the involvement of any patients or members of the public.

### Ethical Considerations

This study was approved by the Ethics Committee of Qingpu Traditional Chinese Medicine Hospital, Shanghai (2024BL0098), and is registered with the International Traditional Medicine Clinical Trial Registry (ITMCTR2024000635). Written informed consent will be obtained from all participants before the commencement of data collection. In the event of adverse reactions during the study, participants will receive appropriate medical care tailored to their specific needs. The findings from this trial will be shared at relevant conferences focusing on KOA, ranging from local to international levels. A detailed manuscript will be prepared and submitted to a peer-reviewed journal for publication, ensuring the results reach the broader scientific community. The primary outcomes will also be communicated directly to participants. Additionally, tailored dissemination efforts, including professional workshops, public lectures, and online platforms, will target researchers, clinicians, and health care providers to maximize the practical application of the study’s findings. This approach will ensure transparency and accessibility, irrespective of the study’s results.

This study does not collect any information that can directly identify the personal identity of participants (such as name, ID number, address, telephone number, etc.). All research data (including clinical scale scores, behavioral test results, and deidentified neuroimaging data) will be identified and processed with study numbers after collection. The data will be stored on a password-protected independent research computer, accessible only to members of our research team with necessary knowledge. When the research results are published, no personal information that may be traced to the participants will be disclosed. Participation in this study is entirely voluntary and does not provide any form of economic reward or compensation to participants.

## Results

This study was funded in August 2024. This experiment is currently not open for recruitment. The experimental plan will begin on December 31, 2025, and will end on May 4, 2027.

## Discussion

### Anticipated Findings

The primary characteristics of KOA are pain, stiffness, and limited mobility, which significantly impair patients’ quality of life and daily activities [53,54]. Pain management constitutes a core component of KOA treatment. On note, patients may continue to experience pain even after surgical intervention, as emphasized in both the Osteoarthritis Research Society International guidelines (2014) [55] and the Chinese Guidelines for the Diagnosis and Treatment of Osteoarthritis (2018) [7]. Tuina and acupuncture have been widely used in the treatment of clinical symptoms, such as pain in KOA [56-59]. Although more evidence is still needed to support the effectiveness of these therapies in relieving KOA symptoms, Chinese Tuina, particularly, as a typical representative of noninvasive and nonpharmacological therapies in traditional Chinese medicine, has received increasing attention and verification for its clinical effects in reducing pain and improving function in patients with KOA [56-60]. Therefore, this study will evaluate the clinical efficacy of acupuncture-combined Tuina in the treatment of KOA, through clinical scale assessment, behavioral assessment, and the application of multimodal neuroimaging techniques. It will also deeply explore its central nervous system analgesic mechanisms, providing more evidence for its clinical application in KOA.

The integration of validated clinical scales and behavioral tests in this trial provides a multidimensional framework for evaluating KOA interventions. The VAS, 36-Item Short Form Health Survey, and WOMAC are accepted to evaluate patients’ pain intensity and life standards. Although subjective in nature, these scales mentioned earlier are widely used and universally recognized treatments [17,18]. Complementarily, the WOMAC index dissects symptom impact into pain, stiffness, and functional domains, offering granularity beyond global pain scores [47]. The inclusion of the WOMAC scale aligns with international guidelines and better reflects the comprehensive clinical assessment of KOA symptoms. In terms of behavioral assessments, the 10MWT is used to quantify walking speed, which serves as a proxy for real-world mobility limitations in patients with KOA [61]. Reduced walking time correlates not only with biomechanical improvement but also with decreased fear of movement, reflecting neuromotor integration [46,53]. Pain threshold (via algometry) and muscle tension measurements target peripheral mechanisms. Elevated pressure pain thresholds may indicate reduced peripheral sensitization, while diminished quadriceps tension suggests reduced muscle coordination—both potentially mediated by descending pain modulation pathways [44,62]. Although this pragmatic study comparing 2 active treatment approaches could not implement blinding for participants and struggled to fully distinguish the specific biomechanical effects of massage from nonspecific factors (such as increased therapeutic attention and contact time), it directly addresses a core question in clinical practice: whether combined massage therapy yields additional benefits in real-world treatment scenarios.

This study used multi-MRI technology to explore the central analgesic brain mechanisms of Tuina intervention for KOA. This approach was chosen due to its advantages of high spatial resolution, absence of radiation, fast imaging speed, and noninvasiveness [63-65], enabling the assessment of brain GM structure, brain function, and WM integrity. KOA pain may be associated with morphological changes in subcortical structures and nuclei, representing part of the neuroimaging pathogenesis [66,67]. DTI can be used to quantify microstructural and macrostructural changes in WM [68]. Functional magnetic resonance imaging allows for in-depth analysis of patients’ pain perception and brain regulatory mechanisms for motor control. It is widely applied in research on the central mechanisms of Tuina analgesia [69-73] and has preliminarily identified functional changes in key brain regions, such as the amygdala, hypothalamus, nucleus accumbens, and hippocampus [74-76].

Current research has confirmed that both acupuncture and Tuina can improve clinical symptoms in patients with KOA, such as pain and dysfunction. The onset of clinical efficacy may be related to improvements in brain functional activity [40,62,77-81]. However, there is a lack of comprehensive, multidimensional research integrating brain GM structure, WM structure, and brain functional behavior for the combined treatment of KOA with Tuina and acupuncture.

The study’s extended follow-up period, including assessments at 12 weeks posttreatment, enhances its design by providing insights into the long-term effects of Tuina and acupuncture for KOA. Rigorous blinding protocols for outcome assessors and statisticians further ensure the reliability and validity of the findings. Unified training for all practitioners and assessors promotes consistency in intervention delivery, data collection, and analysis, thereby enhancing methodological robustness [82].

### Study Strengths and Limitations

This study will be a single-center, strictly randomized controlled clinical trial comparing Tuina combined with acupuncture versus acupuncture alone. For the first time, it will use multi-MRI to explore the central nervous system’s analgesic mechanisms of Tuina combined with acupuncture in treating KOA. The study will implement blinding measures for evaluators and statisticians, and the clinical observation period is relatively long, including a 6-week intervention period and a 12-week follow-up period.

This study has certain limitations. Due to the nature of the therapies, complete blinding of participants and therapists to the treatment assignment was not feasible. More specifically, a key consideration is that both groups received active acupuncture, while the Tuina group received additional manual therapy. This difference in treatment intensity and contact time means that nonspecific factors may contribute to the outcomes observed in the combined treatment group, and their effects cannot be isolated from the specific biomechanical effects of Tuina. This is a recognized challenge in trials comparing complex, multicomponent nonpharmacological interventions. Nevertheless, by implementing blinding of outcome assessors and statisticians and by using objective neuroimaging biomarkers, we have sought to mitigate bias and gain insights into the central effects of the treatment package. The findings from this first-of-its-kind multimodal neuroimaging study will provide crucial preliminary evidence and generate specific hypotheses to guide the design of future, more controlled studies aimed at dissecting the specific mechanisms of Tuina.

### Summary

This randomized controlled trial investigates the efficacy of acupuncture-combined Tuina for treating KOA and explores its neuroimaging-based central analgesic mechanisms, thereby providing a scientific basis for its clinical application.

## Supplementary material

10.2196/84082 Checklist 1 SPIRIT 2025 checklist.

## Abbreviations

10MWT: 10-meter walking test
AE: adverse event
CRF: case report form
DTI: diffusion tensor imaging
GM: gray matter
KOA: knee osteoarthritis
MRI: magnetic resonance imaging
multi-MRI: multimodal magnetic resonance imaging
SF-MPQ: short-form McGill Pain Questionnaire
TE: echo time
TR: repetition time
VAS: visual analog scale
WM: white matter
WOMAC: the Western Ontario and McMaster Universities Osteoarthritis Index
